# Impact of levetiracetam on direct oral anticoagulant level and outcomes among older Asian patients with atrial fibrillation

**DOI:** 10.3389/fphar.2025.1505665

**Published:** 2025-02-19

**Authors:** Yu-Ting Huang, Tzu-Ling Chen, Yun-Lin Huang, Ching-Hua Kuo, Yu-Fong Peng, Sung-Chun Tang, Jiann-Shing Jeng, Chih-Feng Huang, Shin-Yi Lin, Fang-Ju Lin

**Affiliations:** ^1^ Department of Pharmacy, National Taiwan University Hospital, Taipei, Taiwan; ^2^ School of Pharmacy, College of Medicine, National Taiwan University, Taipei, Taiwan; ^3^ Department of Neurology, National Taiwan University Hospital, Taipei, Taiwan; ^4^ Graduate Institute of Clinical Pharmacy, College of Medicine, National Taiwan University, Taipei, Taiwan

**Keywords:** direct oral anticoagulants, levetiracetam, drug interaction, elderly, thromboembolism, bleeding

## Abstract

**Objective:**

This purpose of this study is to analyze the influence of levetiracetam (LEV) on direct oral anticoagulant (DOAC) exposure and its implications for clinical outcomes.

**Methods:**

This investigation comprised a retrospective cohort study utilizing the integrated medical database and a prospective observational study conducted in a tertiary hospital. Patients aged >65 years with atrial fibrillation and undergoing DOAC therapy were included and were categorized as LEV users and non-users based on LEV exposure status. In retrospective cohort, clinical outcomes between LEV users and non-users were compared, included ischemic stroke or transient ischemic attack (IS/TIA), systemic thromboembolism (STE) and major bleeding. In prospective cohort, DOAC trough concentration was measured.

**Results:**

The retrospective study included 191 LEV users and 694 matched LEV non-users. The risk of IS/TIA and STE were not significantly different between two groups (hazard ratio [HR], 0.99 [0.51–1.91] and 0.94 [0.49–1.79], respectively). For major bleeding, a non-significant higher risk was observed in the LEV-user group in contrast to the LEV-non-user group (HR 2.65 [0.43, 16.33]). The prospective analysis included 19 LEV users and 76 matched LEV non-users. Low DOAC concentrations were observed in 5.3% of LEV-users and 14.5% of LEV non-users (*P* = 0.53). High DOAC concentration were observed in 10.5% of LEV-users and 11.8% LEV non-users (P = 0.57). The association between LEV therapy and low or high DOAC concentration was non-significant.

**Conclusion:**

Concurrent use of LEV and DOAC did not significantly affect DOAC exposure or clinical outcomes. LEV may be a safe anti-seizure medication for patients receiving DOAC therapy.

## 1 Introduction

Direct oral anticoagulants (DOACs) are the first-line treatment for stroke in patients with atrial fibrillation (AF). DOACs have fewer drug interactions than vitamin K antagonists and do not require routine coagulation monitoring (Steffel et al., 2021). However, all DOACs are P-glycoprotein (P-gp) substrates, and both apixaban and rivaroxaban undergo metabolism via the cytochrome P450 (CYP) system (Mueck et al., 2014; Byon et al., 2019), highlighting the pharmacokinetic considerations essential for their optimal use. The concurrent use of medications that affect the CYP system or P-gp can alter DOAC metabolism and affect clinical outcomes (Taha et al., 2020).

Post-stroke epilepsy occurs in 3%–6% of patients with stroke, and the risk increases with age (Holtkamp et al., 2017). This condition necessitates the simultaneous management of stroke sequelae and seizure control, often requiring the use of anti-seizure medications (ASMs) alongside DOAC therapy. First-generation ASMs, such as phenytoin, valproic acid, and carbamazepine, are known to interact with CYP enzymes or P-gp (Schmidt and Schachter, 2014). Levetiracetam (LEV), belonging to the newer generation of ASMs, has favorable characteristics including linear pharmacokinetics and fewer drug interactions (Patsalos, 2004), making it an appealing option for managing partial seizures (Privitera, 2001), post-stroke seizure (Belcastro et al., 2011), and seizure in older patients (Lippa et al., 2010). Additionally, LEV is better tolerated than carbamazepine for focal epilepsy in older adults (Werhahn et al., 2015). Despite these benefits, the concurrent use of LEV and DOACs is not without concern. Animal studies have shown that LEV is a weak inducer of P-gp and CYP3A4 (Galgani et al., 2018), potentially leading to decreased DOAC bioavailability. The European Heart Rhythm Association (EHRA) suggests the cautious use of LEV in patients receiving polypharmacy or multiple enzyme-inducing agents (Steffel et al., 2021).

Data regarding the effects of concurrent LEV and DOAC use are limited and conflicting. For instance, a study conducted in Hong Kong indicated that ASMs, including LEV, which modulate CYP enzymes and P-gp, were associated with an increased risk of ischemic stroke in patients undergoing DOAC therapy (Ip et al., 2022). In contrast, an investigation from Taiwan reported an elevated risk of bleeding events in patients concomitantly treated with LEV and DOACs (Wang et al., 2020). Despite these studies, there is a notable gap in real-world evidence related to how LEV influences DOAC plasma concentrations. This study aimed to evaluate the impact of LEV therapy on DOAC concentrations and clinical outcomes, with a specific focus on older patients who are more likely to receive polypharmacy and are more vulnerable to complications.

## 2 Methods

### 2.1 Overview

Our study comprised two parts, each designed to investigate the interactions between LEV and DOACs in older patients with AF. The first was a retrospective cohort study conducted using electronic health records (EHR) from a tertiary medical center. This section specifically examined the clinical outcomes of patients using DOACs and compared them with and without concomitant LEV treatment. The requirement for informed consent was waived owing to the use of de-identified data. The second part was a prospective study focusing on the pharmacokinetic effects of LEV on DOAC concentrations. We included patients who had participated in a registry study on DOAC concentration measurements. Factors related to DOAC concentrations were assessed, with emphasis on the impact of LEV administration. All individuals involved in this registry study provided informed consent before enrollment. The study protocol was approved by the Research Ethics Committee of the National Taiwan University Hospital (No. 201912233RINC, 202101078RINC).

### 2.2 Data source and study cohorts

#### 2.2.1 Cohort for retrospective EHR analysis

Data were retrieved from the integrated medical database of the National Taiwan University Hospital (NTUH-iMD) spanning the period between 1 July 2012, and 31 December 2019. We included older patients (aged ≥65 years) diagnosed with AF (identified through at least one inpatient or two outpatient diagnoses) and who had been prescribed DOACs (dabigatran, rivaroxaban, apixaban, or edoxaban) for more than 3 days. Patients were categorized into groups based on their use of LEV during DOAC treatment: (a) LEV users who were concurrently administered LEV and (b) LEV nonusers who did not receive LEV while on DOAC therapy. The index date was the date of initiating concurrent LEV and DOAC use in LEV users and the date of starting DOAC therapy in LEV nonusers. Continuous use of DOAC or LEV was determined for periods with interruptions between two prescriptions not longer than 14 days. For LEV users, only the first instance of combination therapy with LEV and DOAC was considered for the analysis if the patients had multiple episodes of starting and stopping LEV during their DOAC treatment course.

#### 2.2.2 Cohort with DOAC concentration measurements

To investigate the potential pharmacokinetic interactions between DOAC and LEV, participants were enrolled from the Direct Oral Anticoagulant-Taiwan (DOAC-T) registry established in 2016 (NCT05333666). We included patients with AF aged 65 years or older who were receiving DOAC therapy and collected blood samples between 1 November 2016, and 31 January 2022. The concentration of DOACs was measured at the trough (immediately before the next dose) during the steady state using ultra-performance liquid chromatography-mass spectrometry (UHPLC-MS/MS). The UHPLC-MS/MS method, detailed in Supplementary Material, has been validated and published in previous investigations (Jhang et al., 2020). The DOAC concentrations were evaluated against the established expected therapeutic ranges reported by the EHRA: trough concentrations of 28–215 ng/mL for dabigatran, 12–137 ng/mL for rivaroxaban, 34–230 ng/mL for apixaban, and 12–43 ng/mL for edoxaban (Steffel et al., 2021). The date of concentration measurement served as the index date for the analysis. Based on the LEV exposure status on the index date, the participants were categorized into LEV user and non-user groups. To ensure a robust comparative analysis, each LEV user was matched with up to four LEV-non-users by age (difference of no more than 5 years), sex, and type of DOAC treatment.

### 2.3 Study outcomes and follow-up

The primary clinical outcome was the occurrence of ischemic stroke or transient ischemic attack (TIA). The secondary outcomes included systemic thromboembolism (STE) and major bleeding, the latter defined by the Platelet Inhibition and Patient Outcomes (PLATO) criteria, including the occurrence of intracranial hemorrhage (Abou Kaoud et al., 2023).

The follow-up period for clinical events began on the index date and continued until the earliest of the following: (a) 14 days after the cessation of DOAC and LEV combination therapy in the LEV-user group, or upon the conclusion of DOAC therapy in the LEV-non-user group; (b) occurrence of study outcomes; (c) loss to follow-up; (d) death; or (e) the end of the study period, which was 31 December 2019, for the first part of the study and 31 December 2022, for the second part. This extended follow-up of 14 days after the discontinuation of LEV in the LEV user group was implemented to account for the properties of LEV as a P-glycoprotein inducer, which may continue to affect drug interactions even after the medication has been discontinued (Supplementary Figure S1 in the Supplementary Material).

### 2.4 Statistical analysis

Descriptive statistics were used to summarize the data, including the means, standard deviations, medians, and ranges. Group differences were analyzed using Student’s t-test for continuous variables with a normal distribution, Mann-Whitney U test for continuous variables that were not normally distributed, and the chi-squared test or Fisher’s exact test for categorical variables.

Propensity score (PS) matching was used to balance the potential confounders between LEV users and nonusers in the EHR analysis. Each LEV user was matched to at least four LEV nonusers. The covariates included in the PS matching were age, sex, body mass index, laboratory test results (including renal function, liver function, and hemoglobin A1C), comorbid diseases, CHA_2_DS_2_-VASc score, HAS-BLED score, and concurrent medications. Of note, the item “labile international normalized ratio (INR)” in the HAS-BLED score was omitted because it was not available for DOAC users. Comorbid diseases and laboratory tests were collected within 3 months before the index date. Medical conditions were identified using the International Classification of Diseases, Ninth and Tenth Revisions, Clinical Modification (ICD-9-CM and ICD-10-CM) codes, and the medications were identified using the World Health Organization Anatomical Therapeutic Chemical (ATC) codes.

Logistic regression analysis was conducted to identify the factors associated with DOAC concentrations above or below the expected therapeutic range. Initially, univariate analyses were performed to identify potential variables significantly associated with DOAC concentrations outside the expected range (p < 0.1). Subsequently, the identified variables were incorporated into a multivariate analysis. Creatinine clearance (CrCL) was specifically included because of its recognized influence on DOAC pharmacokinetics, which can affect drug concentrations. Multivariable analysis was used to assess the impact of LEV use on the variability in DOAC concentrations. The Cox proportional hazards model was used to assess the effect of LEV on clinical outcomes, and the proportional hazards assumption was appropriately tested. The Kaplan-Meier curve was presented using the Log-Rank test. All statistical analyses were conducted using SAS software (version 9.4; SAS Institute Inc., Cary, NC, United States) and IBM SPSS Statistics (version 8.0; IBM Corp., Armonk, NY, IBM Corp.). P < 0.05 was set as the threshold for statistical significance.

## 3 Results

### 3.1 Retrospective EHR analysis

**FIGURE 1:**
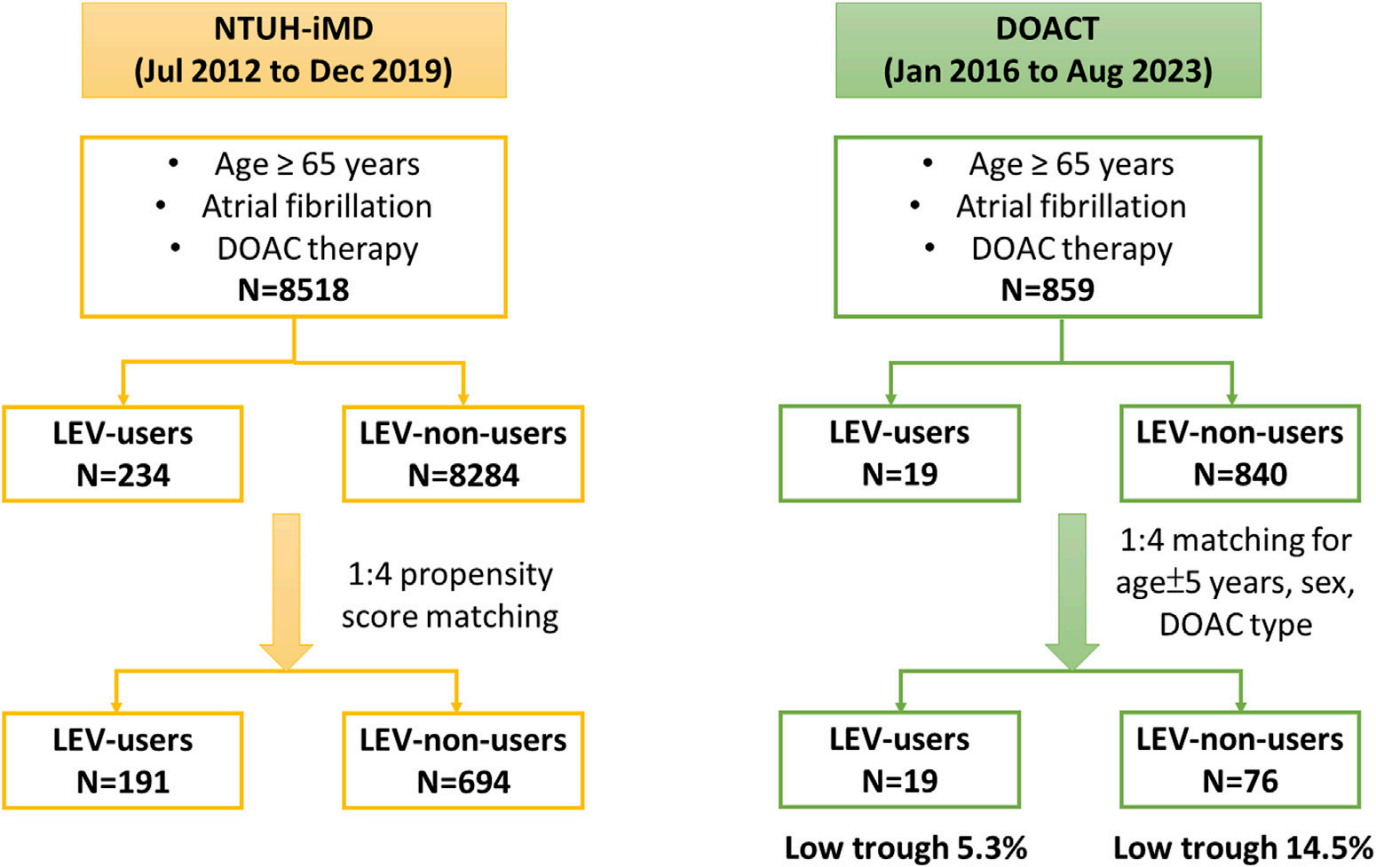
Flow diagram of participant enrollment. Abbreviations: DOAC, direct oral anticoagulant; DOAC-T, the direct oral anticoagulant registry in Taiwan; LEV, levetiracetam; NTUH-iMD, National Taiwan University Hospital integrated medical database.

During the study, 8,752 patients met the inclusion criteria, of whom 234 (2.67%) concurrently used LEV and DOAC. After PS matching, the cohorts were refined to 191 LEV users and 694 LEV nonusers. The study enrollment process is shown in Figure 1. The demographic and clinical characteristics of both groups before and after PS matching are summarized in Table 1. The cohorts were well-balanced. Among medications known to interact with DOACs, the most commonly used were antiarrhythmic agents, particularly amiodarone. A few patients concurrently used ASMs other than LEV, most commonly phenytoin or valproic acid. The proportion of patients using immunosuppressants was very low.

**TABLE 1:** Patient characteristics before and after propensity score matching.

	Before propensity score matching			After propensity score matching		
	LEV-users N = 234	LEV non-users N = 8,284	SMD	LEV-users N = 191	LEV non-users N = 694	SMD^a^
DOAC type			NA			NA
Dabigatran	45 (19.2)	2,585 (31.2)		38 (19.9)	221 (31.8)	
Rivaroxaban	67 (28.6)	2,298 (27.7)		55 (28.8)	157 (22.6)	
Apixaban	93 (39.7)	2,367 (28.6)		71 (37.2)	243 (35.0)	
Edoxaban	29 (12.4)	1,037 (12.5)		27 (14.1)	73 (10.5)	
Age (year)	79.1 ± 7.5	76.6 ± 7.7	0.31	78.7 ± 7.5	78.7 ± 7.6	<-0.01
Male sex	116 (49.6)	4,449 (53.7)	−0.07	89 (46.6)	312 (45.0)	0.02
BMI (kg/m^2^)	23.4 ± 4.4	24.8 ± 4.1	−0.30	23.7 ± 4.5	23.8 ± 4.0	<-0.01
CrCL (mL/min)	44.4 ± 18.1	51.0 ± 18.7	−0.35	44.6 ± 18.4	46.0 ± 18.1	−0.08
Hb_A1C_ (%)	6.3 ± 1.0	6.2 ± 1.0	0.01	6.2 ± 1.0	6.2 ± 1.1	−0.01
Hypertension	186 (79.5)	5,557 (67.1)	0.28	152 (79.6)	542 (78.1)	0.06
Diabetes	86 (36.8)	2,380 (28.7)	0.17	69 (36.1)	250 (36.0)	0.02
Dyslipidemia	98 (41.9)	2,637 (31.8)	0.21	84 (44.0)	302 (43.5)	0.03
CHF	83 (35.5)	2,491 (30.2)	0.12	69 (36.1)	244 (35.2)	−0.01
CAD	63 (26.9)	2,505 (30.2)	−0.08	57 (29.8)	212 (30.6)	−0.01
MI	10 (4.3)	333 (4.0)	0.01	9 (4.7)	34 (4.9)	−0.01
PAOD	18 (7.7)	391 (4.7)	0.13	15 (7.9)	64 (9.2)	−0.04
IS/TIA	134 (57.3)	1,452 (17.5)	0.90	113 (59.2)	412 (59.4)	0
CHA_2_DS_2_-VASc	5.1 ± 1.5	3.8 ± 1.5	0.84	5.1 ± 1.5	5.1 ± 1.6	−0.02
HAS-BLED score ≥3	176 (75.2)	4,142 (50.0)	0.72	129 (67.5)	476 (68.6)	0.04
Concurrent medications						
Antiarrhythmic agents						
Amiodarone	86 (36.8)	2,659 (32.1)	0.07	68 (35.6)	260 (37.5)	−0.04
Dronedarone	3 (1.3)	374 (4.5)		3 (1.57)	23 (3.31)	
Verapamil	7 (3.0)	205 (2.5)	0.08	7 (3.7)	11 (1.6)	−0.04
Diltiazem	38 (16.2)	1,212 (14.6)		27 (14.1)	125 (18.0)	
Antiseizure medications						
Phenytoin	18 (7.7)	16 (0.2)	0.39	6 (3.1)	9 (1.3)	0
Valproic acid	50 (21.4)	53 (0.6)	0.70	22 (11.5)	33 (4.8)	0.02
Carbamazepine	0 (0)	12 (0.14)	0.22	0 (0)	3 (0.43)	0.02
Phenobarbital	0 (0)	1 (0.01)		0 (0)	0 (0)	
Topiramate	3 (1.3)	9 (0.1)		1 (0.52)	6 (0.86)	
Lacosamide	2 (0.9)	1 (0.01)		1 (0.52)	1 (0.14)	
Lamotrigene	2 (0.85)	0 (0)		1 (0.52)	0 (0)	
Immunosuppressants						
Cyclosporine	1 (0.43)	12 (0.14)	0.03	1 (0.5)	1 (0.1)	0.02
Tacrolimus	0 (0)	9 (0.11)		0 (0)	2 (0.3)	
Sirolimus	0 (0)	2 (0.02)		0 (0)	0 (0)	
Antiplatelet agents						
Aspirin	21 (9.0)	1,368 (16.5)	−0.21	17 (8.9)	84 (12.1)	−0.05
Clopidogrel	17 (7.3)	881 (10.6)		16 (8.4)	63 (9.1)	
NSAIDs	60 (25.6)	1,093 (23.0)	0.05	46 (24.1)	172 (24.8)	−0.03

^a^ In some cases, due to small sample sizes, the standard mean difference (SMD) was calculated by combining multiple groups.

Abbreviations: ALT, alanine aminotransferase; BMI, body mass index; CAD, coronary artery disease; CHF, congestive heart failure; CrCL, creatinine clearance; Hb_A1C_, hemoglobin A1C; IS, ischemic stroke; LEV, levetiracetam; MI, myocardial infarction; NA, non-applicable; NSAIDs, non-steroidal anti-inflammatory drugs; PAOD, peripheral arterial occlusive disease; SMD, standardized mean difference; TIA, transient ischemic attack.

**FIGURE 2:**
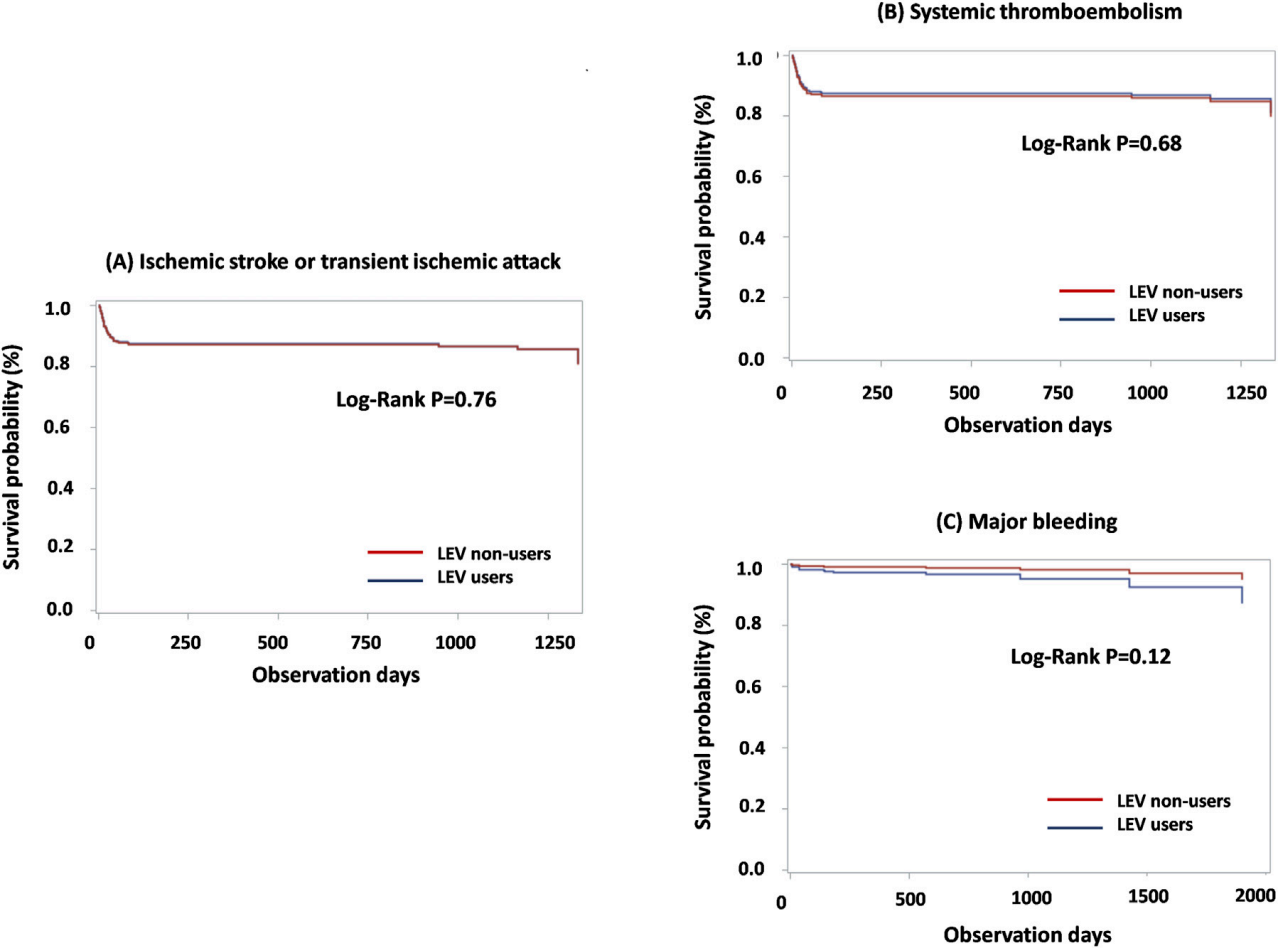
Kaplan-Meier plot for primary and secondary outcomes among matched LEV users and non-users: **(A)** Ischemic stroke or transient ischemic attack; **(B)** Systemic thromboembolism; **(C)** Major bleeding. Abbreviations: LEV, levetiracetam.

The incidence of the primary and secondary outcomes are detailed in Table 2. As the primary outcome, ischemic stroke or TIA was observed in 15 LEV users (18.68 per 100 person-years) and 84 LEV nonusers (11.82 per 100 person-years). The incidence ratio was 1.58 (95% CI: 0.91–2.74). Cox proportional hazards regression analysis revealed a non-significant hazard ratio (HR) of 0.99 (95% CI: 0.51–1.91) for the two groups. Major bleeding events occurred in 3 LEV users (3.65 per 100 person-years) compared with 8 LEV non-users (1.05 per 100 person-years). Cox regression analysis suggested a higher risk in the LEV user group, although the difference was not significant (HR, 2.65; 95% CI: 0.43–16.33). The incidence of STE was not significantly different for the groups (HR, 0.94; 84% CI: 0.49–1.79). The Kaplan-Meier plots for the primary and secondary outcomes, illustrating the cumulative incidence over time, are presented in Figure 2.

**TABLE 2:** The incidence of primary and secondary outcomes.

	Event number	Incidence (per-100-person-years)	Hazard ratio
Primary outcome			
IS/TIA			
LEV-users	15	18.68	0.99 (0.51, 1.91)
LEV-non-users	84	11.82	Reference
Secondary outcomes			
STE			
LEV-users	15	18.68	0.94 (0.49, 1.79)
LEV-non-users	86	12.11	Reference
Major bleeding			
LEV-users	3	3.65	2.65 (0.43, 16.33)
LEV-non-users	8	1.05	Reference

Abbreviations: IS, ischemic stroke; LEV, levetiracetam; STE, systemic thromboembolism; TIA, transient ischemic attack.

### 3.2 DOAC concentration measurements

A total of 859 patients enrolled in the DOAC-T registry met the inclusion criteria. Following matching, the final cohort included 19 LEV users and 76 LEV non-users. The basic characteristics of the two groups are presented in Table 3. Similar to the retrospective cohort, the most commonly used medication with known interactions with DOACs was amiodarone. The proportion of patients with lower-than-expected DOAC concentrations was 5.3% for the LEV users and 14.5% for the non-users. However, this difference was not statistically significant (*P* = 0.53). Multivariate logistic regression adjusted for clinical variables showed that LEV therapy was not a significant predictor of lower-than-expected trough concentrations (adjusted odds ratio [aOR]: 0.36 [95% CI: 0.04, 3.01], *P* = 0.34) (Table 4; Supplementary Table S1). Conversely, the proportion of patients with higher-than-expected DOAC concentrations was 10.5% for the LEV user group and 11.8% for the LEV non-user group, with no significant difference between the groups (*P* = 0.57, Table 4). LEV was also not a significant factor in predicting higher-than-expected trough concentrations (aOR: 2.45 [95% CI: 0.46, 13.07], Supplementary Table S1).

**TABLE 3:** Basic characteristics for patients with or without concurrent use of levetiracetam.

Characteristics	LEV-users n = 19	LEV-non-users n = 76	p-value
Age (year)	78.0 ± 8.3	78.2 ± 8.2	0.94
Male	9 (47.4)	36 (47.4)	1.00
Body mass index (kg/m^2^)	24.8 ± 4.2	24.3 ± 4.2	0.67
CrCl (mL/min)	48.1 ± 18.2	51.1 ± 20.8	0.78
CHA_2_DS_2_VASc score	5.1 ± 1.2	4.3 ± 1.6	**0.03**
HAS-BLED score	2.8 ± 0.6	2.4 ± 0.8	**0.03**
Ischemic stroke/TIA history	14 (73.7)	32 (42.1)	**0.02**
Hypertension	17 (89.5)	56 (73.7)	0.22
Diabetic mellitus	4 (21.1)	23 (30.3)	0.43
Congestive heart failure	3 (15.8)	15 (19.7)	1.00
Coronary artery disease	5 (26.3)	13 (17.1)	0.35
History of ICH	2 (10.5)	2 (2.6)	0.18
History of GI bleeding	0 (0)	6 (7.9)	0.34
Dose regimen^a^			
Standard dose	4 (21.1)	32 (42.1)	0.12
Reduced dose	15 (78.9)	44 (57.0)	
Trough level (ng/mL)			
Dabigatran (n = 5)	88.4	165.7 ± 111.3	0.48
Rivaroxaban (n = 25)	71.0 ± 64.8	63.2 ± 81.5	0.25
Apixaban (n = 50)	100.2 ± 64.4	117.3 ± 63.5	0.29
Edoxaban (n = 15)	35.6 ± 31.1	28.9 ± 25.8	0.67
Lower than expected range	1 (5.3)	11 (14.5)	0.53
Higher than expected range	2 (10.5)	9 (11.8)	
Concurrent medications^b^			
Amiodaroone	1 (5.3)	14 (18.4)	0.29
Dronedarone	1 (5.3)	7 (9.2)	1.00
Aspirin	0 (0)	1 (1.3)	1.00
Clopidogrel	0 (0)	1 (1.3)	1.00
NSAIDs	0 (0)	1 (1.3)	1.00

Data are expressed as mean ± standard deviation or number (percentage). Bold number indicates p-value reaches the level of significance.

^a^ Standard dose regimen is defined as dabigatran 150 mg twice daily, rivaroxaban 15 mg daily (according to the labeling in Taiwan), apixaban 5 mg twice daily and edoxaban 60 mg daily. Other doses lower than the standard dose regimen is defined as reduced dose regimen.

^b^ Due to the small sample size, most patients did not concurrently use interacting drugs. Therefore, only the interacting drugs used by patients were listed. Abbreviations: BMI, body mass index; CrCl, creatinine clearance; GI, gastrointestinal; ICH, intracranial hemorrhage; LEV, levetiracetam; TIA, transient ischemic attack.

**TABLE 4:** Factors associated with lower-than-expected-range trough level.

Factor	Multivariate analysis^a^	
	Odds ratio	P-value
Age (year)	0.95 (0.86, 1.06)	0.33
CrCl (mL/min)	1.02 (0.98, 1.05)	0.30
Levetiracetam	0.36 (0.04, 3.01)	0.34

Abbreviations: CrCL, creatinine clearance.

The incidences of the primary and secondary outcomes are shown in Supplementary Table S2. Ischemic stroke or TIA occurred in one patient in the LEV user group relative to three patients in the LEV non-user group (risk rate ratio: 1.06 [95% CI: 0.02–13.19]). For secondary outcomes, five patients had STE (one LEV user and four LEV non-users, risk rate ratio: 0.79 [0.02–7.94]), and nine patients had major bleeding (two LEV users and seven LEV non-users, risk rate ratio: 0.90 [0.09–4.71]).

## 4 Discussion

This study represents the first investigation of the concurrent use of LEV and DOAC and its impact on DOAC exposure and clinical outcomes among older Asian patients with AF. Our findings indicate that the concurrent administration of LEV and DOAC does not significantly affect the risk of IS/TIA, STE, major bleeding, or altered DOAC exposure.

Few studies have focused on the drug interactions between DOAC and LEV because of the limited population treated with this combination. Data from the Taiwanese insurance research database enrolled the largest number of patients (approximately 10,592 LEV users compared to 721,131 LEV non-users) (Wang et al., 2020). However, IS/TIA and STE were not analyzed in that study. Additionally, the findings of this investigation contradict the known mechanism behind this interaction. As a P-gp inducer, LEV theoretically reduces DOAC exposure and subsequently increases the risk of thromboembolism (Steffel et al., 2021).

Several other investigations have addressed this topic; however, the sample sizes were relatively limited. An Israeli investigation with a nested case-control design based on an insurance database showed that LEV was associated with stroke or STE risk; however, the LEV users and non-users were few (9 and 74, respectively). Additionally, this study enrolled patients who used DOAC for AF or deep vein thrombosis. Therefore, these results may not be completely generalized to patients with AF (Gronich et al., 2021). Another study analyzing data from the Israeli Food and Drug Administration Adverse Event Reporting System (FAERS) reported increased odds of anticoagulant treatment failure in patients treated with rivaroxaban or apixaban who were concurrently using enzyme-inducing ASMs, including LEV (Abou Kaoud et al., 2023). However, adverse events associated with the FAERS are self-reported, and the incidence of thromboembolic events may not be precisely estimated.

From our data, the proportion of patients with low DOAC trough concentrations was not significantly different for LEV users and non-users, indicating a lack of significant effect of LEV on the pharmacokinetic properties of DOAC. P-gp-mediated induction by LEV has only been observed in vivo animal studies (Steffel et al., 2021; Mathy et al., 2019). In a phase I study, concurrent use of LEV and digoxin, a P-gp substrate, in healthy human participants did not alter the pharmacokinetic or pharmacodynamic properties of digoxin (Levy et al., 2001). Therefore, the effects of LEV on P-gp in animals cannot be directly extrapolated to humans. LEV remains a safe option in patients under DOAC who require ASMs.

This study concentrated on the interaction between LEV and DOACs, specifically examining both clinical outcomes and DOAC exposure concentrations. By simultaneously investigating the effect of this drug combination on thromboembolism and bleeding events, our research provides comprehensive insights into the safety profile of this drug combination in older Asian patients with AF. Despite its strengths, this study had several limitations that must be acknowledged. First, DOAC exposure was assessed using trough concentrations rather than serial measurements across dosing intervals. This approach limited our ability to evaluate the influence of LEV on the area under the concentration-time curve for DOACs. Future studies should employ population pharmacokinetic analyses to provide a more detailed assessment of this interaction. Second, the impact of different LEV doses on DOAC interactions was not assessed due to the limited sample size. Additionally, in the DOAC-T cohort with concentration measurements, the small patient number made the findings inconclusive, especially dabigatran users. Further research with a larger cohort would allow for subgroup analyses to determine whether the extent of drug interactions varies across different dosing regimens of LEV. Third, there is a potential immortal time bias in our study design. Patients in the LEV user group may have been on relatively more stable DOAC treatment regimens than LEV non-users. In addition, differences between the follow-up durations of LEV users and non-users can lead to biased estimates of clinical outcome rates. Lastly, the impact of genetic polymorphisms on DOAC exposure was not evaluated in the present study. Genes encoding P-glycoprotein, such as *ABCB1* (ATP-Binding Cassette Sub-Family B Member 1), can influence DOAC exposure. Although some studies have reported that the *ABCB1* genotype is not a significant determinant of inter-individual variability in the pharmacokinetics of dabigatran and rivaroxaban (Gouin-Thibault et al., 2017), this remains an important concern requiring further investigation.

## 5 Conclusion

This study represents a pioneering effort to simultaneously investigate DOAC exposure and clinical outcomes associated with the concurrent use of LEV and DOACs in older patients with AF. Contrary to expectations based on the pharmacological profile of LEV as a P-glycoprotein inducer, our findings indicated that LEV does not significantly alter DOAC exposure or affect the incidence of ischemic stroke, STE, or major bleeding events. These results suggest that LEV can be safely co-administered with DOACs in this patient population without necessitating adjustments to DOAC dosing. However, given the limitations of our study, further research using larger and more diverse cohorts and detailed pharmacokinetic profiling is essential to fully elucidate the clinical implications of this drug interaction.

## Data Availability

The raw data supporting the conclusions of this article will be made available by the authors, without undue reservation.
