# Supplementary material for: Impact of levetiracetam on direct oral anticoagulant level and outcomes among older Asian patients with atrial fibrillation
Source: Front Pharmacol. 2025 Feb 19;16:1505665. doi: 10.3389/fphar.2025.1505665 (PMC11880207; doi:10.3389/fphar.2025.1505665)
Supplement: Supplementary file 1 [file Supplementaryfile1.docx]

Supplementary Material

# The method of Ultra-high-performance liquid chromatography with tandem mass spectrometry conditions for direct oral anticoagulant concentration measurement

Blood samples were collected through venous puncturing and stored in tubes containing K2EDTA (BD Vacutainer®), and were centrifuged with a standard procedure to obtain plasma and stored in −80°C until use. Plasma NOAC concentration was measured by using ultra-high performance liquid chromatography with tandem mass spectrometry (UHPLC-MS/MS). In total, 100 µL of each plasma sample was deproteinized by adding 800 μL of 100% MeOH and homogenized at 1000 rpm for 2 min in a Geno/Grinder 2010 (SPEX SamplePrep; Metuchen, NJ, USA), followed by centrifugation at 15,000 rpm for 5 min. Thereafter, 400 µL of the supernatant was transferred to a new Eppendorf tube. The plasma extracts were dried with a centrifugal vaporizer (Thermo SpeedVac® Savant SPD111V; Thermo Fisher Scientific, Waltham, MA, USA), followed by reconstitution of the sample in 200 μL of MeOH. The reconstituted sample was then filtered through a 0.22-μm polypropylene membrane filter (RC-4; Sartorius, Göttingen, Germany) for injection into a system for ultra-high performance liquid chromatography with tandem mass spectrometry (UHPLC-MS/MS). The instrument used was an Agilent 1290 UHPLC system coupled with an Agilent 6460 triple quadrupole mass spectrometer (Agilent Technologies, Santa Clara, CA, USA). A Kinetex reversed-phase core-shell C18 column (2.1 × 50 mm, 2.6 µm, 100 Å; Phenomenex, Torrance, CA, USA) was used for separation. The mobile phase consisted of 0.1% formic acid and 10 mM ammonium acetate in water (solvent A), and 0.1% formic acid and 10 mM ammonium acetate in isopropanol and ACN (9:1, v/v) (solvent B). The flow rate was 0.35 mL min-1. The gradient profile started with 0% B for 0.5 min, then changed to 6% B in 0.1 min and remained at 6% B for 0.6 min, subsequently increased to 25% B in 0.5 min, 27.5% B in 0.5 min, and 50% B in 0.5 min, and was maintained 50% B for 1 min. Finally, the column was re-equilibrated to 0% B for 2 min until the next injection. The temperature of the sample reservoir was maintained at 4°C, and the column oven temperature was set to 55°C. The injection volume was 3 µL. A JetStream electrospray ionizer was employed as the ion source. The MS parameters were set as follows: a 350°C drying gas temperature, 10 L/min drying gas flow rate, 45-psi nebulizer pressure, 350°C sheath gas temperature, an 11-L/min sheath gas flow rate, a 3500-V capillary voltage, and a 500-V nozzle voltage. Mass spectrometry (MS) data acquisition was executed in multiple reaction monitoring mode, and the mass transitions were 472.2→289, 472.2→144 for dabigatran, 478.2→295.1, 478.2→144 for [13C6]-dabigatran, 436.1→144.9, 436.1→72.9 for rivaroxaban, 442.1→144.9, 442.1→72.9 for [13C6]-rivaroxaban, 460.2→443.1, 460.2→199 for apixaban, 464.2→447.1, 464.2→203.1 for [13C, d3]-apixaban, 548.1→366.1, 548.1→152 for edoxaban, 554.1→372.1, 554.1, 158.1 [d6]-edoxaban.

# Supplementary Tables

**Table S1.** Factors associated with out-of-expected-range trough level.

| **Trough lower than expected range** | | | | |
| --- | --- | --- | --- | --- |
| **Factor** | **Univariable analysis** | | **Multivariable analysis** | |
|  | **Odds ratio** | **P-value** | **Odds ratio** | **P-value** |
| Age (year) | 0.92 (0.85, 1.01) | **0.08** | 0.95 (0.86, 1.06) | 0.33 |
| Male sex | 1.13 (0.34, 3.79) | 0.85 |  |  |
| CrCl (mL/min) | 1.03 (1.00, 1.06) | **0.04** | 1.02 (0.98, 1.05) | 0.30 |
| CHA_2_DS_2_-VASc score | 0.90 (0.60, 1.36) | 0.62 |  |  |
| HAS-BLED score | 1.31 (0.60, 2.87) | 0.50 |  |  |
| Ischemic stroke / TIA history | 1.08 (0.32, 3.61) | 0.91 |  |  |
| Hypertension | 0.55 (0.15, 2.05) | 0.38 |  |  |
| Diabetic mellitus | 1.98 (0.57, 6.89) | 0.28 |  |  |
| Congestive heart failure | 0.35 (0.04, 2.93) | 0.34 |  |  |
| Standard dose regimen^1^ | 0.80 (0.22, 2.86) | 0.73 |  |  |
| Levetiracetam | 0.33 (0.04, 2.72) | 0.30 | 0.36 (0.04, 3.01) | 0.34 |
| Amiodarone | 0.45 (0.05, 3.76) | 0.46 |  |  |
| Dronedarone | 2.57 (0.46, 14.49) | 0.29 |  |  |
| Antiplatelet agent^2^ | Non-applicable |  |  |  |
| **Trough higher than expected range** | | | | |
| **Factor** | **Univariable analysis** | | **Multivariable analysis** | |
|  | **Odds ratio** | **P-value** | **Odds ratio** | **P-value** |
| Age (year) | 0.90 (0.81, 1.00) | **0.04** | 0.93 (0.82, 1.05) | 0.25 |
| Male sex | 0.92 (0.26, 3.24) | 0.89 |  |  |
| CrCl (mL/min) | 1.02 (0.99, 1.05) | 0.27 | 0.99 (0.96, 1.03) | 0.76 |
| CHA_2_DS_2_-VASc score | 0.83 (0.54, 1.28) | 0.40 |  |  |
| HAS-BLED score | 0.61 (0.27 1.40) | 0.24 |  |  |
| Ischemic stroke / TIA history | 0.87 (0.25, 3.09) | 0.83 |  |  |
| Hypertension | 0.48 (0.13, 1.81) | 0.28 |  |  |
| Diabetic mellitus | 1.52 (0.41, 5.67) | 0.54 |  |  |
| Congestive heart failure | 2.86 (0.74, 11.09) | 0.13 |  |  |
| Standard dose regimen^1^ | 5.33 (1.31, 21.68) | **0.02** | 3.35 (0.64, 17.60) | 0.15 |
| Levetiracetam | 0.88 (0.17, 4.43) | 0.87 | 1.18 (0.20, 6.96) | 0.85 |
| Amiodarone | 0.50 (0.06, 4.23) | 0.52 |  |  |
| Dronedarone | 1.10 (0.12, 9.89) | 0.93 |  |  |
| Antiplatelet agent | 8.30 (0.48, 143.26) | 0.15 |  |  |

^1^Standard dose regimen: dabigatran 150 mg twice daily, rivaroxaban 15 mg daily, apixaban 5 mg twice daily, or edoxaban 60 mg daily. Any dose regimen lower than the standard dose regimen is the reduced dose regimen. In our hospital, rivaroxban 20 mg tablet was not available, hence all participants used rivaroxaban according to the J-ROCKET AF dosing regimen. The low dose regimen group is the reference group.

**Abbreviations:** CrCL, creatinine clearance; TIA, transient ischemic attack.

**Table S2.** The incidence of primary and secondary outcomes.

|  | Event number | Incidence  (per-100-person-years) | Incidence rate ratio  (95% CI) |
| --- | --- | --- | --- |
| **Primary outcome** |  |  |  |
| **IS/TIA** |  |  |  |
| LEV-users | 1 | 1.74 | 1.06 (0.02, 13.19) |
| LEV-non-users | 3 | 1.64 |  |
| **Secondary outcomes** |  |  |  |
| **STE** |  |  |  |
| LEV-users | 1 | 1.74 | 0.79 (0.02, 7.94) |
| LEV-non-users | 4 | 2.22 |  |
| **Major bleeding** |  |  |  |
| LEV-users | 2 | 3.49 | 0.90 (0.09, 4.71) |
| LEV-non-users | 7 | 3.89 |  |

Abbreviations: CI, confidence interval; LEV, levetiracetam.


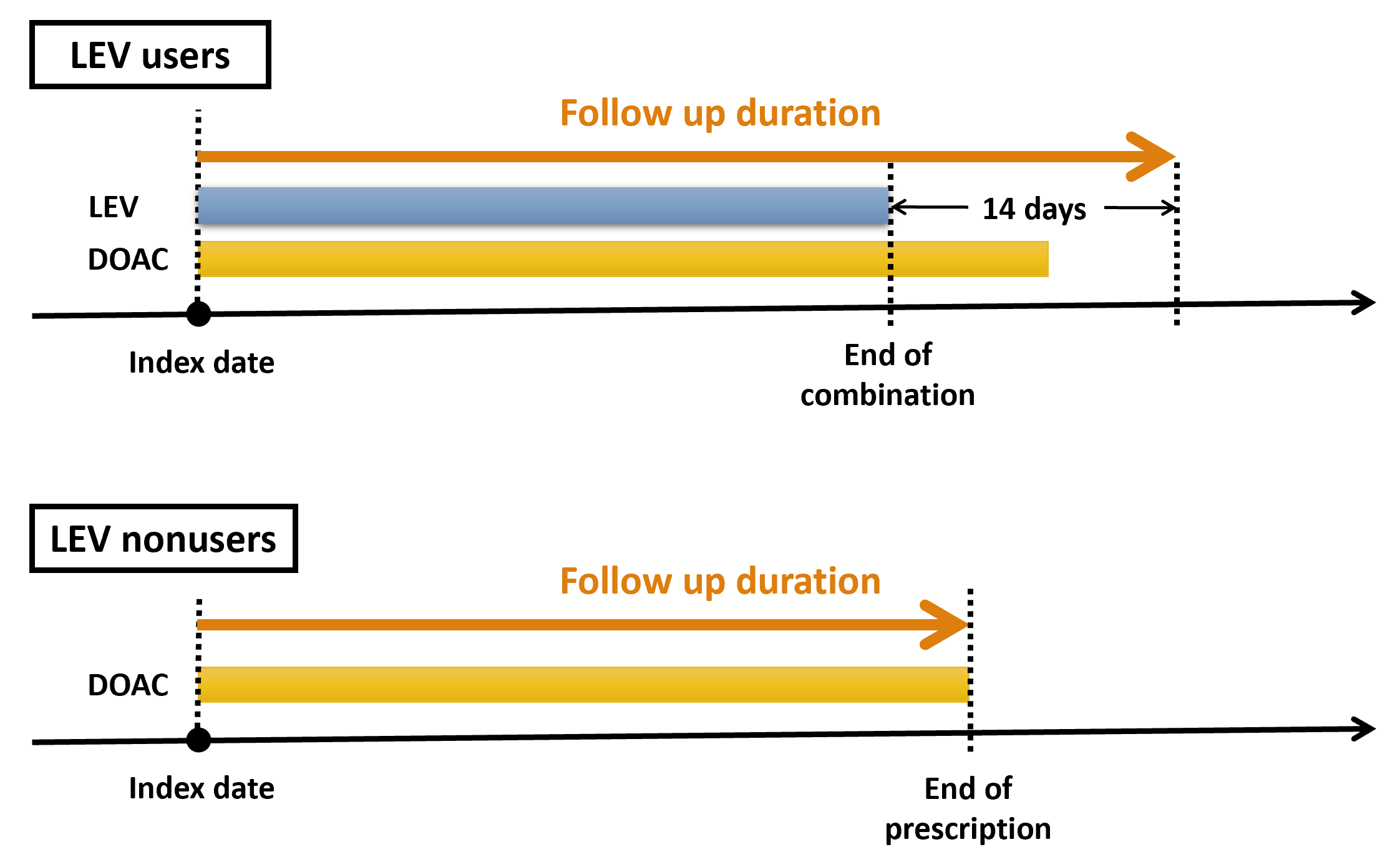


**Figure S1.** The diagraph of follow up duration between levetiracetam users and nonusers.
